# Management strategies for vaccinated animals after an outbreak of foot-and-mouth disease and the impact on return to trade

**DOI:** 10.1371/journal.pone.0223518

**Published:** 2019-10-11

**Authors:** Richard Bradhurst, Graeme Garner, Iain East, Clare Death, Aaron Dodd, Tom Kompas

**Affiliations:** 1 Centre of Excellence for Biosecurity Risk Analysis, University of Melbourne, Parkville, VIC, Australia; 2 Epidemiology and One Health, Animal Health Policy Branch, Department of Agriculture and Water Resources, Canberra, ACT, Australia; Pirbright Institute, UNITED KINGDOM

## Abstract

An incursion of Foot-and-mouth disease (FMD) in a previously FMD-free country can cause significant economic damage from immediate and prolonged closure of FMD-sensitive markets. Whilst emergency vaccination may help contain disease, the presence of vaccinated animals complicates post-outbreak management and the recovery of FMD-free status for return to trade. We present enhancements to the Australian Animal DISease (AADIS) model that allow comparisons of post-outbreak management strategies for vaccinated animals, for the purposes of securing the earliest possible return to trade. Two case studies are provided that compare the retention of vaccinated animals with removal for waste/salvage, and the impact on recovery of FMD-sensitive markets per OIE guidelines. It was found that a vaccinate-and-retain strategy was associated with lower post-outbreak management costs, however this advantage was outweighed by significantly higher trade losses. Under the assumptions of the study there was no cost advantage to salvaging the removed vaccinated animals.

## Introduction

Foot-and-mouth disease (FMD) is recognised as the single greatest disease threat to Australian livestock industries [1]. The present value of total direct economic losses from a large outbreak of FMD in Australia has been estimated at up to A$52.2 billion (Australian dollars) over 10 years [2]. More than 90% of the economic impact of an FMD outbreak in Australia would arise from revenue losses caused by the immediate and prolonged closure of FMD sensitive markets [2]. The Australian policy for an FMD response is to contain, control and eradicate the disease in order to re-establish FMD-free status as quickly as possible, while minimising social and financial disruption. Following an outbreak of FMD, the re-establishment of export markets for affected industries would be one of the highest priorities of disease response efforts [3]. Australia would need to satisfy trading partners that international animal health guidelines for establishing proof-of-freedom have been met. This includes minimum time periods since the last case of disease, and surveillance aimed at identifying disease and FMD infection or transmission [4].

Emergency vaccination is increasingly being recognised as a potential management option during an FMD outbreak. Vaccination may help suppress the spread of infection [5] and reduce the need for large-scale culling of at-risk animals [6]. Modelling studies in Australia [7–9] and internationally [10–14], have indicated that vaccination may be effective in reducing the duration and/or size of FMD outbreaks, particularly when disease is widespread, there is a high rate of spread, or resources for stamping out are limited. It has been suggested that FMD outbreaks in Korea and Japan may have been more effectively controlled with an earlier deployment of vaccination [15–16].

Whilst vaccination may positively contribute to earlier eradication of disease, its use adds complexity to post-outbreak management. A serological survey, based on the detection of antibodies to non-structural proteins of FMD virus, will be required to demonstrate no evidence of infection in the remaining vaccinated population [4]. Further, if vaccinated animals are retained in the population after an outbreak, trade losses will be extended due to the increased waiting period for FMD-free status recovery [17]. Under World Organisation for Animal Health (OIE) guidelines a country can apply to regain FMD-free status three months after the disposal of the last culled animal or the slaughter of all vaccinated animals, whichever occurred last. However, if vaccinated animals are retained in the population then countries must wait at least six months after the detection of the last case or the last vaccination (whichever occurred last), before applying for official freedom from FMD [4].

An emergency vaccination policy for a previously FMD-free jurisdiction should thus take into consideration how vaccinated animals will be managed after an outbreak. Management options are:

- retention in the population to live out normal commercial lives (i.e., a ‘vaccinate-and-retain’ policy)
- removal from the population for disposal (i.e., a ‘vaccinate-and-remove-to-waste’ policy)
- removal from the population for salvage (i.e., a ‘vaccinate-and-remove-for-salvage’ policy)

A vaccinate-and-retain policy avoids large-scale culling of vaccinated animals but complicates the post-outbreak surveillance required to support the recovery of FMD-free status [18]. As vaccinated animals exposed to infection may become sub-clinically and persistently infected it is necessary to find and remove all infected vaccinated animals in order to regain FMD–free status [6, 19]. The retention of vaccinated animals in the population also increases the mandatory waiting period until recovery of FMD-free status [4]. A vaccinate-and-remove policy simplifies post-outbreak surveillance and expedites the recovery of FMD-free status, however the destruction of vaccinated animals has associated economic and social impacts. Some of the removal and processing costs may be offset by the residual salvage value of raw or processed products.

From a policy perspective it can be very useful if disease managers have access to decision support tools that can be used to evaluate policies on, and approaches to, recovery of FMD-free status. The Australian Animal DISease (AADIS) model [20] is a national-scale epidemiological model used by animal health authorities in Australia to support FMD planning and preparedness. In this paper we describe model enhancements to assist with the evaluation of different post-outbreak management strategies with a view to the earliest possible recovery of FMD-free status and return to trade. We provide two case studies that compare retention and removal of vaccinated animals after hypothetical FMD outbreaks in the Australian states of Victoria and Western Australia.

## Materials and methods

### The AADIS epidemiological model

The AADIS model is a spatiotemporal agent-based simulation of the spread and control of emergency animal disease, initially developed by the Australian Government Department of Agriculture and Water Resources in collaboration with the University of New England [20]. (Note that in a modelling context, the term ‘agent’ refers to the modelling unit of interest [21], not an infectious agent.) Each herd agent has an embedded set of differential equations that model the herd’s infected, infectious, serological and clinical prevalence over time, taking into account species, production system and virus strain. For this study we have assumed a Type O Pan-Asia strain. The agents interact in a model environment that stochastically spreads disease across multiple spread pathways (direct contacts, indirect contacts, saleyard spread, airborne transmission, and local spread). Control measures (stamping out, surveillance, tracing, movement restrictions, and vaccination), are implemented per the Australian Veterinary Emergency Plan (AUSVETPLAN) for FMD [3]. Similar to an actual outbreak response, the simulated control measures are dynamically constrained by: the available resources (responders and consumables such as vaccine); the accuracy of reports of clinical disease (as false reports still consume surveillance resources); inefficiencies in tracing systems; and non-compliance with movement restrictions.

AADIS models emergency vaccination inside either user-defined annuli around infected premises (IPs), or within the local government areas in which IPs are located. Vaccination can be further restricted to specific user-defined high-risk areas. The vaccination program is highly configurable as to the trigger (a fixed control day or only when a certain outbreak criteria is reached); the targeted herd types; the direction of vaccination inside the annulus (outside-in or inside-out); and retrospectivity (all IPs or newly declared IPs only). The delivery of the vaccination program is constrained by the time taken to vaccinate the targeted herds, the number of available vaccination teams, and the available supply of vaccine [20].

It is possible for control teams to inadvertently spread infection whilst managing IPs or implementing vaccination programs. In Australia, this risk is well-recognised and addressed through stringent biosecurity and disinfection measures for people and equipment leaving IPs and other premises within restricted areas, and the use of stand-down periods for key personnel that may have been exposed to infection. Under Australia’s Veterinary Emergency Plan, there is a full operational procedures manual covering decontamination [22]. Accordingly, we have assumed that the risk of control teams spreading FMD infection in Australia is negligible, and as such, was not included in the study.

### Modelling post-outbreak management

A new AADIS post-outbreak management module was developed to simulate serological surveillance to support proof-of-freedom, retention/removal of vaccinated animals, compensation for destroyed animals, and loss of trade. All post-outbreak activities are costed (from user-configurable values), and this allows different post-outbreak management strategies to be evaluated in relative economic terms. The module implements three post-outbreak management options for vaccinated animals: vaccinate-and-retain, vaccinate-and-remove-to-waste and vaccinate-and-remove-for-salvage. Some potential advantages and disadvantages of the three strategies are summarised in Table 1. For this study we focussed on measurable costs, and while recognising the importance of intangible factors (such as animal welfare and social acceptability), they are not included in the analysis.

**Table 1:** Advantages and disadvantages of different management strategies for FMD vaccinated animals.

Strategy	Advantages	Disadvantages
Vaccinate-and-retain	No costs of removing animals (slaughter, disposal, compensation) No loss of genetics Continuity of production Producer goodwill Vaccinated animals have a value More public acceptance	Longer time to achieve FMD-free status Delayed return to markets Additional surveillance costs Separate slaughter and product processing chains to safeguard exports to FMD-free markets Additional product processing costs Record keeping and information management Finding new markets
Vaccinate-and-remove-to-waste	Shorter time to achieve FMD-free status Earlier return to markets	Compensation for mandatory acquisition and slaughter of stock Vaccinated animals have no value Animal processing and disposal costs Production loss Loss of genetics Producer resentment Less public acceptance
Vaccinate-and-remove-for-salvage	Shorter time to achieve FMD-free status Earlier return to markets Vaccinated animals may have some residual value	Compensation for mandatory acquisition and slaughter of stock Production loss Loss of genetics Producer resentment Animal processing costs Additional product processing costs Finding new markets

A vaccinate-and-retain strategy allows vaccinated animals to live out their normal commercial lives. As such, the destruction and disposal of vaccinated animals is not undertaken and compensation is not available. There is a six-month waiting period for regaining FMD-free status after the detection of the last case or the last vaccination [4], and comprehensive post-outbreak surveillance of the vaccinated population is undertaken.

Under a vaccinate-and-remove-to-waste strategy all vaccinated animals are destroyed and disposed to waste as part of the post-outbreak management program. Decontamination of premises is not required as the herds were not infected. Full compensation is assumed to be paid as the animals are compulsorily destroyed. There is a three-month waiting period for regaining FMD-free status after the disposal of the last vaccinated animal [4].

A vaccinate-and-remove-for-salvage strategy requires only the destruction of vaccinated animals, with disposal costs assumed to be met by the processor undertaking the salvage. As vaccinated animals are assumed to have a value arising from the sale of salvageable products, compensation is only payable for the difference between the market prices of vaccinated and unvaccinated animals.

The AADIS vaccination option determines the combination of management actions applied to vaccinated animals after an outbreak (Table 2). The default option of Vaccination-disabled (i.e., stamping out only), is included for comparison.

**Table 2:** Modelled aspects of post-outbreak management of vaccinated animals.

Strategy	Surveillance	Destruction	Disposal	Disinfection	Compensation	OIE waiting period
Vaccination disabled	No	No	No	No	No	3 months
Vaccinate-and-retain	Yes	No	No	No	No	6 months
Vaccinate-and-remove to-waste	No	Yes	Yes	No	Full	3 months
Vaccinate-and-remove-for-salvage	No	Yes	No	No	Partial	3 months

### Cost estimates and assumptions

Estimated vaccination costs per animal (Table 3) were based on values from [7] and K. de Witte (personal communication, 2011).

**Table 3:** FMD vaccination cost estimates per head by species.

Species	Labour (A$)	Vaccine (A$)	Cold storage and handling (A$)	Total cost (A$)
Cattle	3.00	1.20	0.80	5.00
Sheep	3.00	0.60	0.80	4.40
Pigs	3.00	1.20	0.80	5.00
Other^†^	3.00	1.20	0.80	5.00

^†^any species present in a smallholder herd (defined as less than 50 animals on less than 20 hectares)

Table 4 summarises the modelled costs for post-outbreak destruction and disposal of, and compensation for, vaccinated animals. Destruction costs were estimated from [7] taking into account inflation. Disposal costs were based on an average disposal cost of A$90 per tonne and average live weights (adults plus juveniles), of 750 kg, 100 kg and 25 kg for cattle, pigs and sheep respectively. Per tonne costs for rendering, deep burial and incineration were drawn from [23], inflated and exchanged into 2017 Australian dollars. This estimate is within the range of reported costs of deep burial in Australia [24], although, it is likely that in some locations prices may be much higher. Compensation payable to owners of vaccinated animals destroyed during the post-outbreak management phase was assumed to be the average market value (adults plus juveniles).

**Table 4:** Cost estimates for post-outbreak management of FMD vaccinated animals per head by species.

Species	Destruction (A$)	Disposal (A$)	Compensation for waste (A$)	Compensation for salvage (A$)
Cattle (intensive/extensive)	6.00	67.50	742.50	371.25
Cattle (feedlot)	6.00	67.50	872.00	436.00
Cattle (dairy)	6.00	67.50	895.00	447.50
Sheep	4.00	2.25	69.00	34.50
Pigs	4.00	9.00	222.50	111.25
Other^†^	3.00	2.25	150.00	75.00

^†^any species present in a smallholder herd

Salvage options for vaccinated animals include: export to non-FMD sensitive markets; domestic consumption; processing for export or domestic consumption; or processing (via a knackery) for pet food. Of these options, slaughter for domestic consumption was considered to be the most feasible (and hence, likely) option for processing the anticipated volume of animals requiring salvage. This is primarily due to the relatively limited capacity of knackery and canning processors, and the difficult technical barriers to trade (such as volume caps and shelf-life limits) in the FMD-endemic meat market [25]. This in turn would likely limit the rate at which animals could be slaughtered and salvaged. The study assumed that vaccinated animals would be salvaged for domestic consumption and the pet food market, at a discount of 50% [7, 26]. Compensation costs were thus reduced by 50% when vaccinated animals were salvaged (Table 4).

It has been estimated that during an FMD outbreak the loss of export markets and the oversupply of animal products on the domestic market would reduce the gross value of production by approximately A$32.5 million per day [2]. The number of days out of market is modelled as the period from the declaration of the index case through to the end of the waiting period for recovery of FMD-free status. Total trade losses from an outbreak were calculated as simply the number of days out of market multiplied by the daily trade loss of A$32.5 million.

Under vaccinate-and-remove strategies, the OIE waiting period does not start until the last vaccinated animal is destroyed [4], thus for each additional day that the commencement of that waiting period is delayed, a loss of A$32.5 million was assumed to be incurred. The trade losses arising from an outbreak are therefore dependent on the time taken to remove vaccinated animals from the population. Estimated removal rates of vaccinated animals to waste were derived from industry reports [27–29], and are provided in Table 5. Salvage removal rates were assumed to be 20% lower to account for increased processing requirements, possible delays in the sale of vaccinated animals, and/or the use of smaller scale processors such as knackeries and canneries.

**Table 5:** Estimated removal rate of FMD vaccinated animals (head per day).

Species	Removal rate to waste (head/day)	Removal rate for salvage (head/day)
Cattle	1000	800
Sheep	5000	4000
Pigs	2000	1600
Other^†^	2600	2080

^†^any species present in a smallholder herd

### Case studies

Two case studies involving hypothetical FMD outbreaks in the Australian states of Victoria (VIC) and Western Australia (WA) are provided below.

#### Victoria outbreak scenario

**Fig 1:**
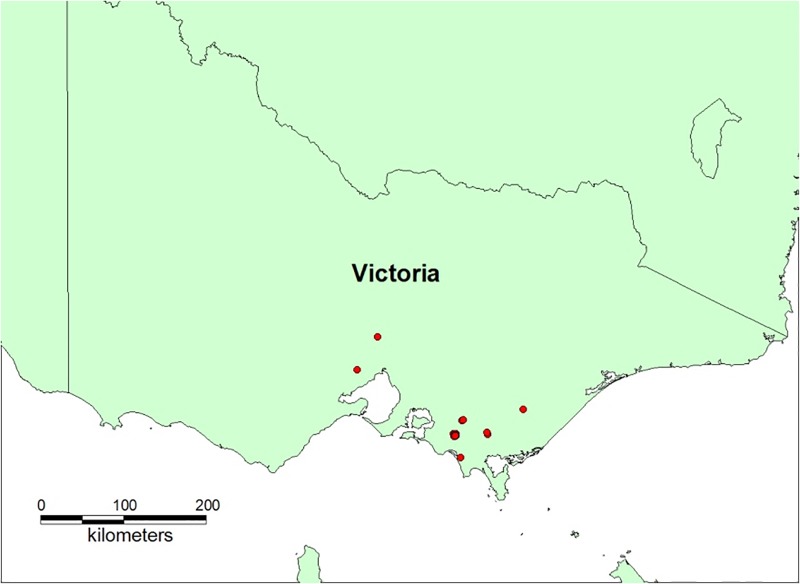
Infected herds (n = 18) in the simulated VIC FMD outbreak scenario at the end of the 14-day silent spread period.

FMD virus of type O was assumed to be introduced into a piggery through illegal feeding of swill containing infectious material sourced from a country where FMD is endemic. The outbreak began in September in a small 25-sow pig farm (n = 247) to the west of Leongatha in the Gippsland region of Victoria (38.4740° S, 145.9437° E). FMD was recognised and reported to the authorities 14 days after introduction, at which point there were 18 infected herds (Fig 1).

The scenario was run using AADIS ‘snapshot seeding’ which is a means of ensuring that each iteration of a simulation has the same initial set of infected herds on the day of first detection. This was achieved by initially running 1000 iterations of just the ‘silent spread’ phase of the VIC outbreak, i.e., from day 0 through to day 14. A single iteration, consistent with the median number of infections from the 1000 runs, was selected as the ‘snapshot’ (Fig 1). This served as a common starting point for the control programs under test (from day 15 onward), for all runs in the case study. Selected configuration parameter settings for the VIC case study are provided in Table 6.

**Table 6:** Control parameter settings for the VIC FMD case study.

Component	Description
Movement restrictions	3-day national livestock standstill Control Area (CA) initially the whole of state of Victoria reducing to a 10 km radius around each IP after 14 days Restricted Area (RA) initially a 10 km radius around each IP reducing to 3 km after 14 days
Stamping out	Culling of all susceptible animals on confirmed IPs only
Vaccination	Emergency vaccination (3 km radius around IPs) of cattle (plus sheep on mixed cattle-sheep properties only) Commences on the 14^th^ day of control program Applied only in previously identified high risk regions Vaccination around new IPs only (i.e., no retrospective vaccination)
Surveillance and tracing	Investigation of all reported suspect premises Periodic visits to premises in RA Tracing of direct and indirect movements onto and off IPs
Resources	Three surveillance teams available at the start of the control program ramping up a maximum of 50 teams over a 3-week period Two culling teams available at the start of the control program ramping up to a maximum of 25 teams over a 4-week period Five disposal teams available at the start of the control program ramping up to a maximum of 40 teams over a 30-day period Vaccination carried out by lay vaccinators (i.e., not constrained by the availability of veterinarians)

#### Western Australia outbreak scenario

**Fig 2:**
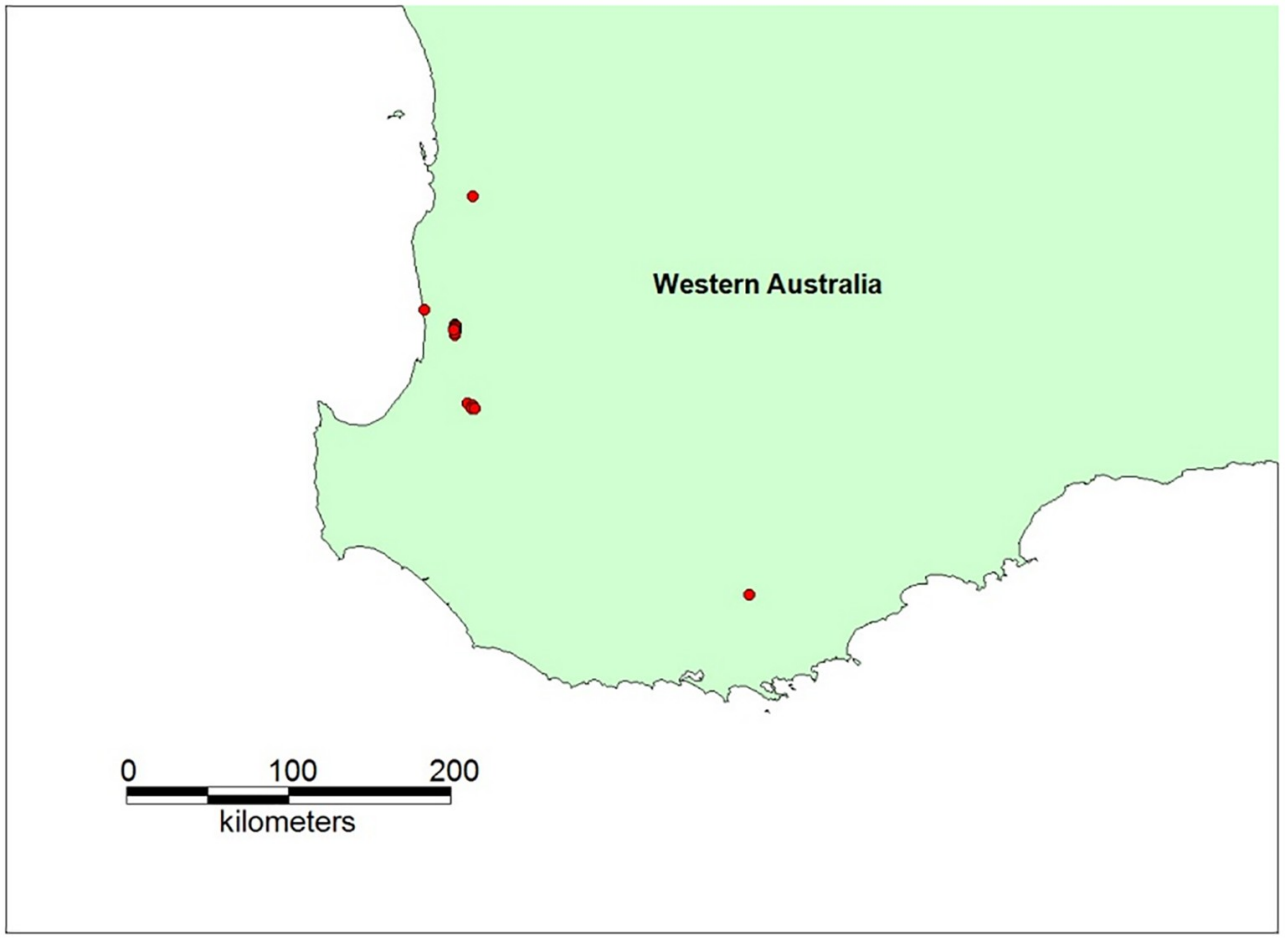
Infected herds (n = 20) in the simulated WA FMD outbreak scenario at the end of the 14-day silent spread period.

FMD virus of type O was assumed introduced via the port of Fremantle, WA (32.0518° S, 115.7551° E). Contaminated imported stockfeed was sent to, and infected, a sheep flock (n = 288) on a mixed sheep-beef farm near the town of Harvey in south-west WA (33.0823° S, 115.9057° E). The outbreak started in May and was detected after 30 days, at which point there were 20 infected herds (Fig 2). (AADIS snapshot seeding was employed to achieve this as described in Section 2.4.1). Selected configuration parameter settings for the WA case study are provided in Table 7

**Table 7:** Control parameter settings for the WA FMD case study.

Component	Description
Movement restrictions	3-day national livestock standstill CA initially the whole of state of Western Australia reducing to a 25 km radius around each IP after 14 days, and a 10 km radius after 28 days RA initially the whole of the local government area enclosing each IP, reducing to a 10 km radius around each IP after 14 days, and a 3 km radius after 28 days
Stamping out	Culling of all susceptible animals on confirmed IPs only
Vaccination	Emergency vaccination (3 km radius around IPs) of cattle (plus sheep on mixed cattle-sheep properties only) Commences on the 14^th^ day of control program Applied only in previously identified high risk areas Vaccination around new IPs only (i.e., no retrospective vaccination)
Surveillance and tracing	Investigation of all reported suspect premises Periodic visits to premises in RA Tracing of direct and indirect movements onto and off IPs
Resources	Four surveillance teams available at the start of the control program ramping up to a maximum of 30 teams over a 3-week period One culling team available at the start of the control program ramping up to a maximum of 20 teams over a 4-week period Four disposal teams available at the start of the control program ramping up to a maximum of 22 teams over a 30-day period Vaccination carried out by lay vaccinators (i.e., not constrained by the availability of veterinarians)

### Study design and statistical methods

One thousand iterations of the VIC and WA case studies were run initially with a stamping out only control program, and then with stamping out plus emergency vaccination, for each of the three vaccination management strategies. For each simulation, the model was run until the control and post-outbreak management programs were completed, or for 365 days, whichever came first.

The effectiveness of vaccination was first assessed by comparing the size and duration of outbreaks with and without vaccination, for each case study.

The vaccinate-and-retain, vaccinate-and-remove-to-waste and vaccinate-and-remove-for-salvage strategies were compared, using the following model outputs:

- Number of IPs
- Duration of the control program (the period in days from the declaration of the index case through to the lifting of the last CA/RA)
- Cost of the control program (including surveillance, culling, disposal, disinfection, compensation, vaccination, and control centres)
- Cost of the post-outbreak management program (including surveillance, laboratory tests, and where applicable, removal of vaccinated animals and compensation)
- Days out of market (from the declaration of the index case through to fulfillment of the OIE waiting periods)
- Trade losses (incurred during the time out of market)
- Total outbreak costs (summation of control costs, post-outbreak management costs and trade losses)

Where comparisons between different control approaches were made, data were analysed using STATA statistical software package [30]. Initially all data were examined for normality using: (i) visual appraisal of histograms of the data, (ii) determination of the skew and kurtosis of each dataset and its deviation from the values expected in a normal distribution, and (iii) an automated search of a subset of the ladder of powers for a transform that converted the data to normality. All of the datasets were non-normal. No standard transformations transformed the data into a normal distribution. All datasets were log transformed to minimise the over-distribution (left skew) observed.

Datasets were compared using both the one-way ANOVA and the Kruskal-Wallis tests. The one-way ANOVA has been reported as robust to deviations from normality when the datasets are large and was used because it is a more rigorous test [31, 32]. In addition, pairwise comparisons of datasets could be done automatically when more than two groups were being compared. To examine the impact of the data being non-normal, all results were checked using the Kruskal-Wallis test. The one-way ANOVA and the Kruskal-Wallis test produced comparable results for each comparison examined. After statistical analysis was complete, summary figures were reverse transformed (antilog) and presented as means with a 95% confidence interval (CI).

RStudio [33] was used to produce box and whisker plots. The box represents the 25–75 percentile range. The horizontal band within the box represents the median. The whiskers represent the 0–25 and 75–100 percentile ranges. The y-axes are presented logarithmically.

## Results

### Case study 1 (VIC)

**Fig 3:**
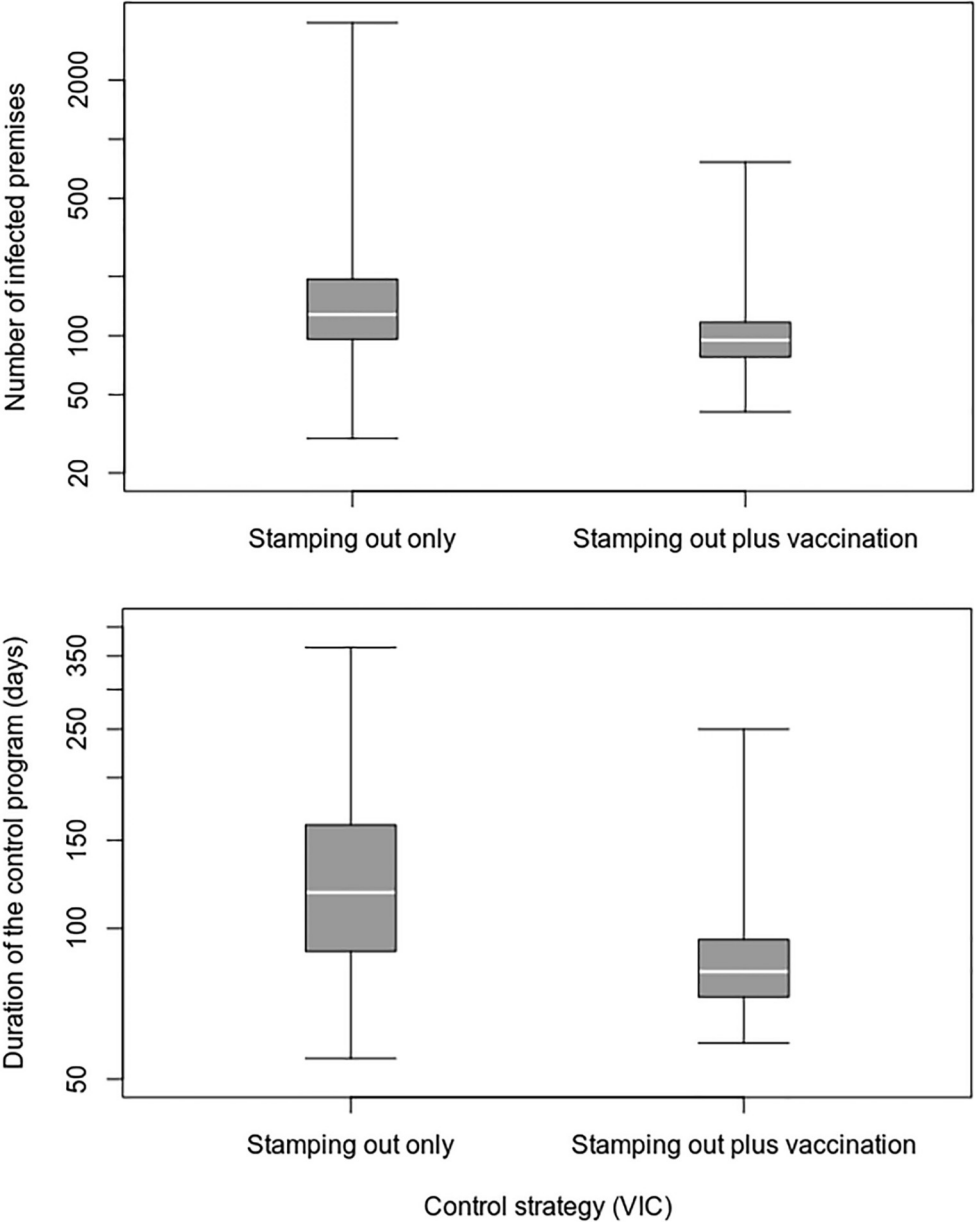
Comparison of vaccination and non-vaccination control strategies for the VIC FMD outbreak scenario.

The inclusion of emergency vaccination in the VIC control program resulted in statistically significant improvements in the effectiveness and cost of control. On average, the duration of the control program was decreased by 39 days (approximately a 31% reduction), the number of IPs by 46 (32%), the number of culled animals by 11,074 (29%), and the cost of the control program by A$21.5 million (32%) (Table 8). Vaccination was effective in reducing outbreak variability and the likelihood of a very large outbreak (Fig 3). Under a stamping out only control strategy, in 24 of the 1000 runs (2.4%), the outbreak was still active at the end of the 365-day simulation period. When vaccination was used, all outbreaks were controlled within the simulation period.

**Table 8:** Statistical summary of vaccination and non-vaccination control strategies for the VIC FMD outbreak scenario.

	Stamping out only mean (95% CI)	Stamping out plus vaccination mean (95% CI)
Number of IPs	144.1 (138.8, 149.5)^a^^†^	98.3 (96.2, 100.4)^b^
Outbreak duration (days)	123.6 (120.6, 126.7)^a^	84.7 (83.6, 85.7)^b^
Cost of control program (A$ million)	67.44 (65.29, 69.67)^a^	45.90 (45.03, 46.79)^b^
Number of animals culled	38728 (37318, 40191)^a^	27654 (26986, 28338)^b^

^†^ Within rows, figures with differing superscripts are significantly different (p < 0.05)

**Fig 4:**
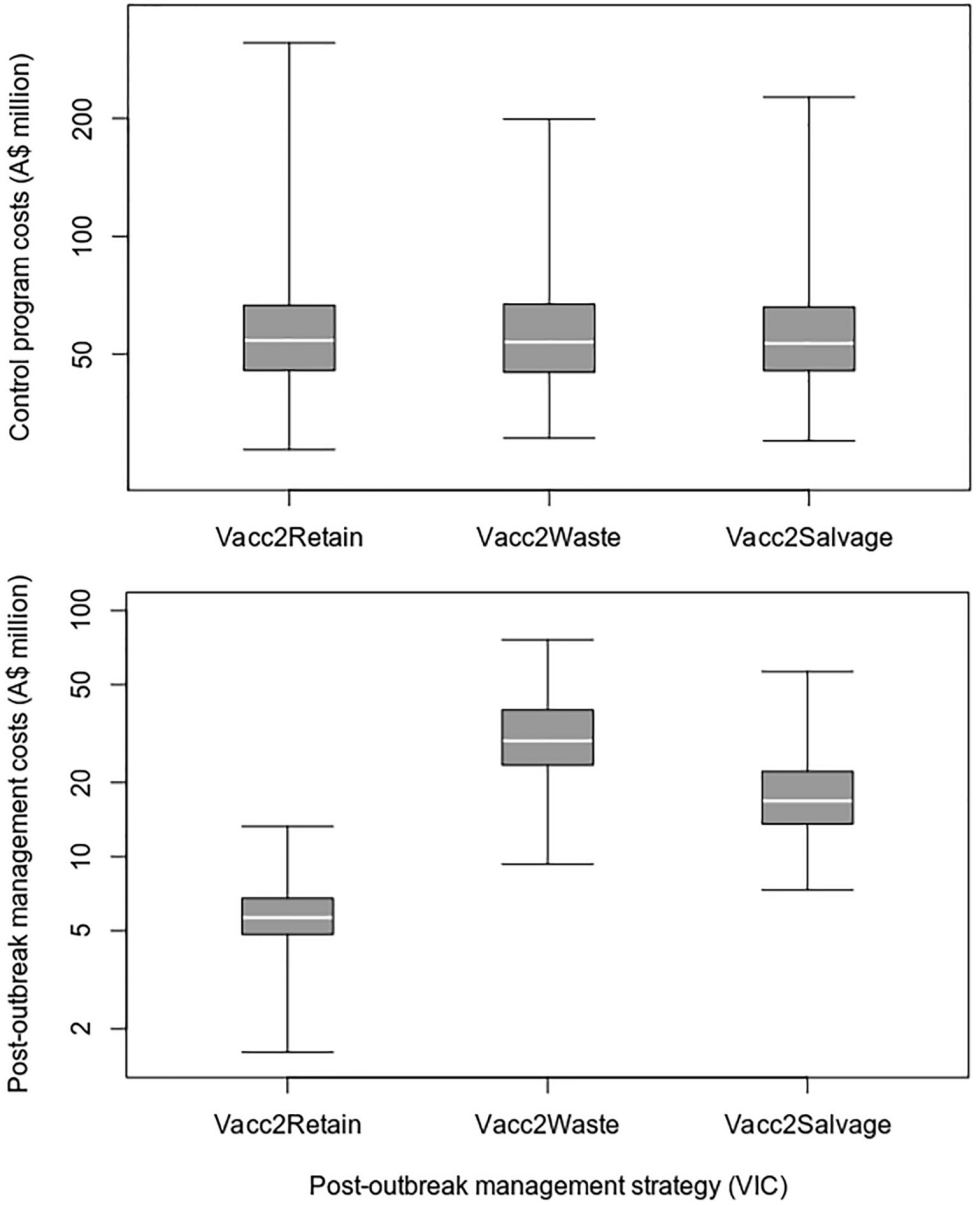
Control and post-outbreak managements costs for different post-outbreak management strategies in the VIC FMD outbreak scenario.

**Fig 5:**
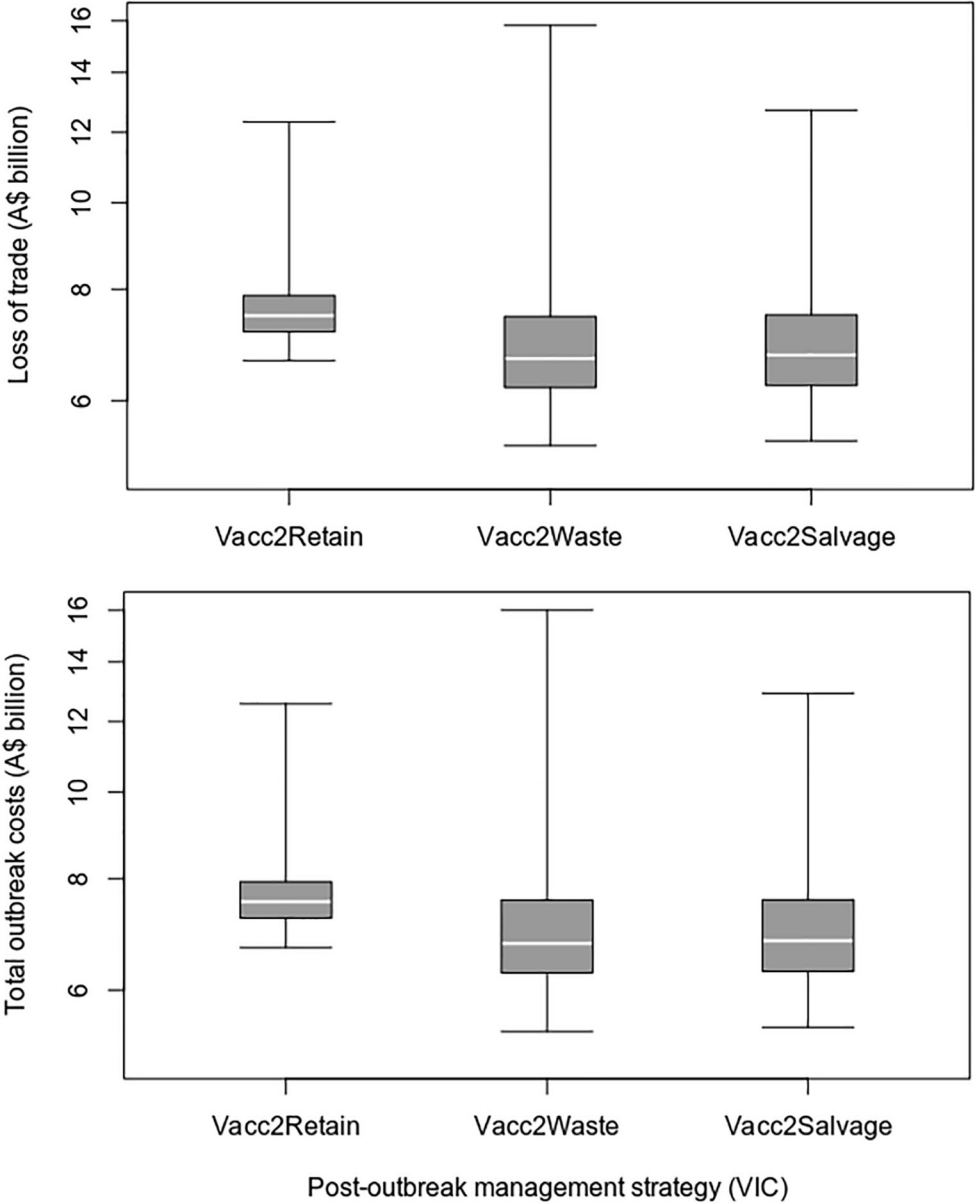
Loss of trade and total outbreak costs for different post-outbreak management strategies in the VIC FMD outbreak scenario.

Under the assumptions used for this case study, the removal of vaccinated animals resulted in higher post-outbreak management costs and lower loss of trade costs, when compared to a vaccinate-and-retain strategy (Figs 4 and 5 and Table 9). On average, the vaccinate-and-remove-to-waste strategy resulted in 21 fewer days out of market and overall savings of A$664 million (8.7%), compared to the vaccinate-and-retain strategy. There was no overall cost benefit from salvaging the removed vaccinated animals.

**Table 9:** Statistical summary of different post-outbreak management strategies for the VIC FMD outbreak scenario.

Model outcome variable	Vaccinate-and-retain mean (95% CI)	Vaccinate-and-remove-to-waste mean (95% CI)	Vaccinate-and-remove-for-salvage mean (95% CI)
Number of IPs	98.7 (96.7, 100.7)^a^^†^	98.1 (96.1, 100.1)^a^	98.9 (96.9, 100.9)^a^
Number of vaccinated premises (VPs)	193.1 (188.2, 198.1)^a^	191.2 (186.4, 196.1)^a^	194.7 (189.8, 199.8)^a^
Control program duration (days)	86.9 (85.9, 88.0)^a^	86.1 (85.1, 87.1)^a^	86.6 (85.6, 87.6)^a^
Surveillance costs (A$ million)	1.38 (1.35, 1.40)^a^	1.36 (1.34, 1.39)^a^	1.38 (1.35, 1.40)^a^
Culling, disposal & decontamination costs (A$ million)	5.58 (5.46, 5.70)^a^	5.54 (5.42, 5.67)^a^	5.61 (5.48, 5.73)^a^
Compensation costs (A$ million)	17.23 (16.82, 17.65)^a^	17.16 (16.75, 17.59)^a^	17.40 (16.97, 17.83)^a^
Vaccination costs (A$ million)	0.20 (0.20, 0.21)^a^	0.20 (0.20, 0.21)^a^	0.20 (0.20. 0.21)^a^
Control centre costs (A$ million)	31.41 (30.92, 31.90)^a^	31.15 (30.64, 31.66)^a^	30.88 (30.40, 31.37)^a^
**Total cost of control (A$ million)**	**56.31 (55.32, 57.33)**^**a**^	**55.98 (54.98, 57.01)**^**a**^	**55.98 (54.98, 57.01)**^**a**^
Post outbreak surveillance duration (days)	42.1 (41.5, 42.7)^a^	40.3 (39.7, 40.8)^b^	40.5 (39.9, 41.1)^b^
Post outbreak surveillance costs (A$ million)	2.94 (2.90, 2.99)^a^	2.93 (2.89, 2.98)^a^	2.95 (2.91, 3.00)^a^
Post outbreak laboratory costs (A$ million)	2.83 (2.79, 2.87)^a^	2.53 (2.50, 2.56)^b^	2.54 (2.50, 2.57)^b^
Post outbreak culling, disposal and decontamination costs (A$ million)	0.00 (0.00, 0.00)^a^	2.07 (2.02, 2.12)^b^	0.00 (0.00, 0.00)^a^
Post outbreak compensation costs (A$ million)	0.00 (0.00, 0.00)^a^	23.05 (22.49, 23.62)^b^	11.99 (11.69, 12.31)^c^
**Total post outbreak management costs** **(A$ million)**	**5.77 (5.69, 5.86)**^**a**^	**30.62 (29.95, 31.30)**^**b**^	**17.63 (17.25, 18.01)**^**c**^
Days out of market	236.6 (235.5, 237.7)^a^	215.2 (213.3, 217.1)^b^	216.6 (214.7, 218.5)^b^
Loss of trade (A$ million)	7575.32 (7540.12, 7610.68)^a^	6886.47 (6826.06, 6947.42)^b^	6931.25 (6870.92, 6992.92)^b^
**Total outbreak cost (A$ million)**	**7639.21 (7603.07, 7675.52)**^**a**^	**6975.21 (6913.32, 7037.65)**^**b**^	**7006.36 (6943.97, 7069.32)**^**b**^

^†^ Within rows, figures with differing superscripts are significantly different (p < 0.05)

### Case study 2 (WA)

**Fig 6:**
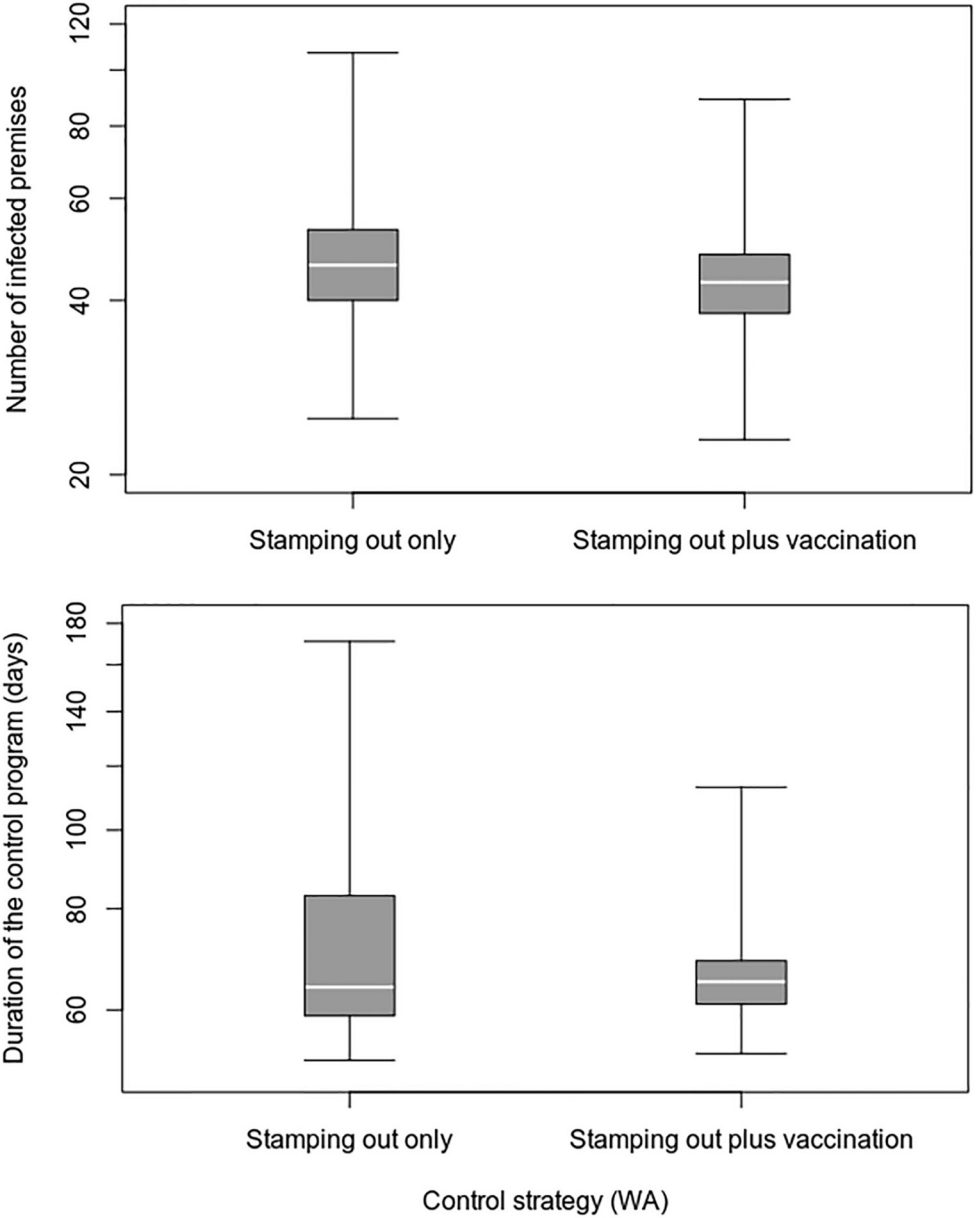
Comparison of vaccination and non-vaccination control strategies for the WA FMD outbreak scenario.

The inclusion of emergency vaccination in the WA control program resulted in small but statistically significant improvements in the effectiveness and cost of control. On average, the duration of the control program was decreased by 6 days (approximately an 8% reduction), the number of IPs by 3 (7%), the number of culled animals by 786 (5%), and the cost of the control program by A$2.3 million (8%) (Table 10). Vaccination was effective in reducing outbreak variability and the likelihood of a large outbreak (Fig 6).

**Table 10:** Statistical summary of vaccination and non-vaccination control strategies for the WA FMD outbreak scenario.

	Stamping out only mean (95% CI)	Stamping out plus vaccination mean (95% CI)
Number of IPs	46.3 (45.7, 47.0)^a^^†^	42.9 (42.4, 43.4)^b^
Outbreak duration (days)	71.8 (70.7, 72.9)^a^	65.7 (65.2, 66.2)^b^
Cost of control program (A$ million)	29.70 (29.30, 30.10)^a^	27.44 (27.22, 27.67)^b^
Number of animals culled	16282 (16026, 16543)^a^	15497 (15267, 15731)^b^

^†^ Within rows, figures with differing superscripts are significantly different (p < 0.05)

**Fig 7:**
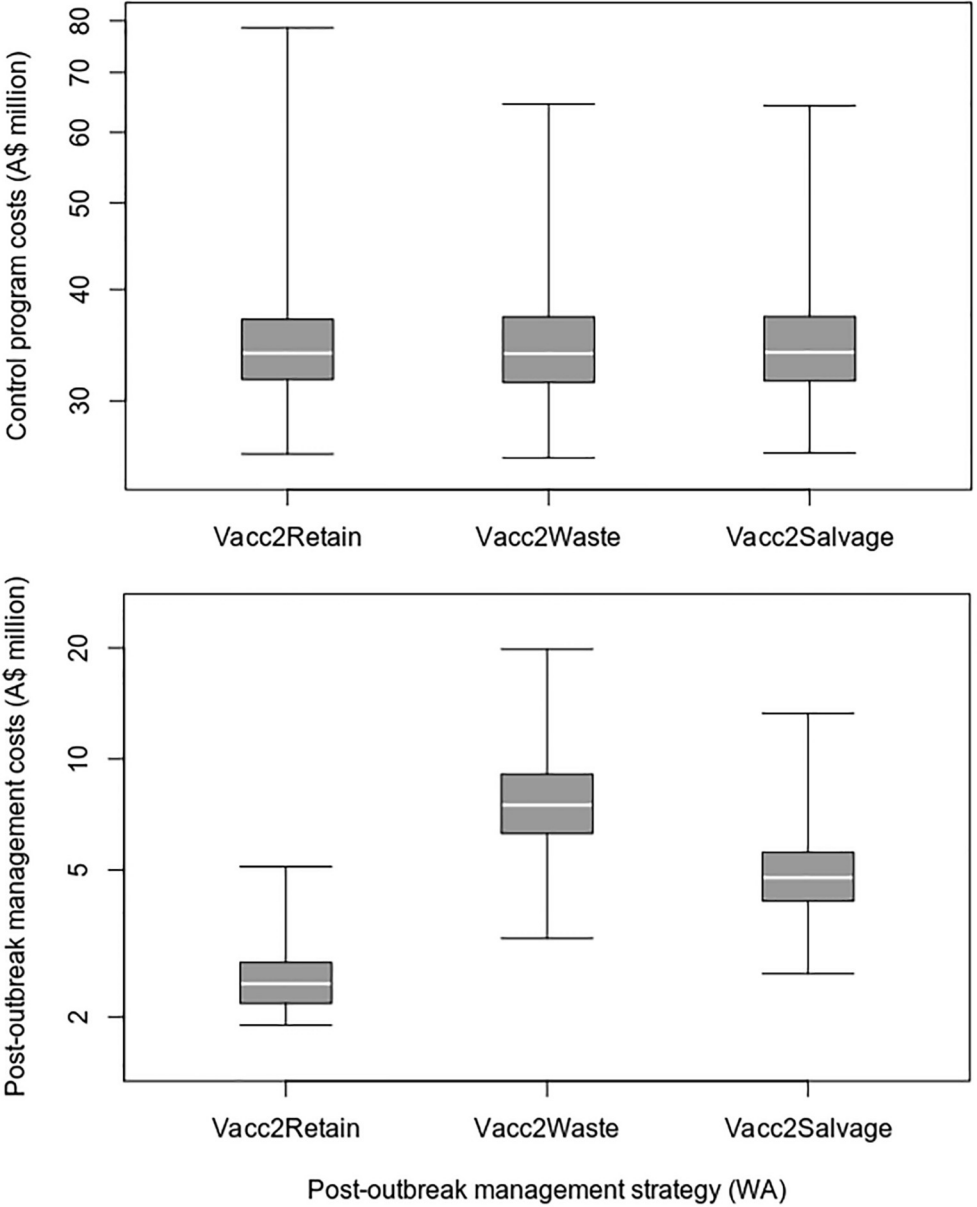
Control and post-outbreak managements costs for different post-outbreak management strategies in the WA FMD outbreak scenario.

**Fig 8:**
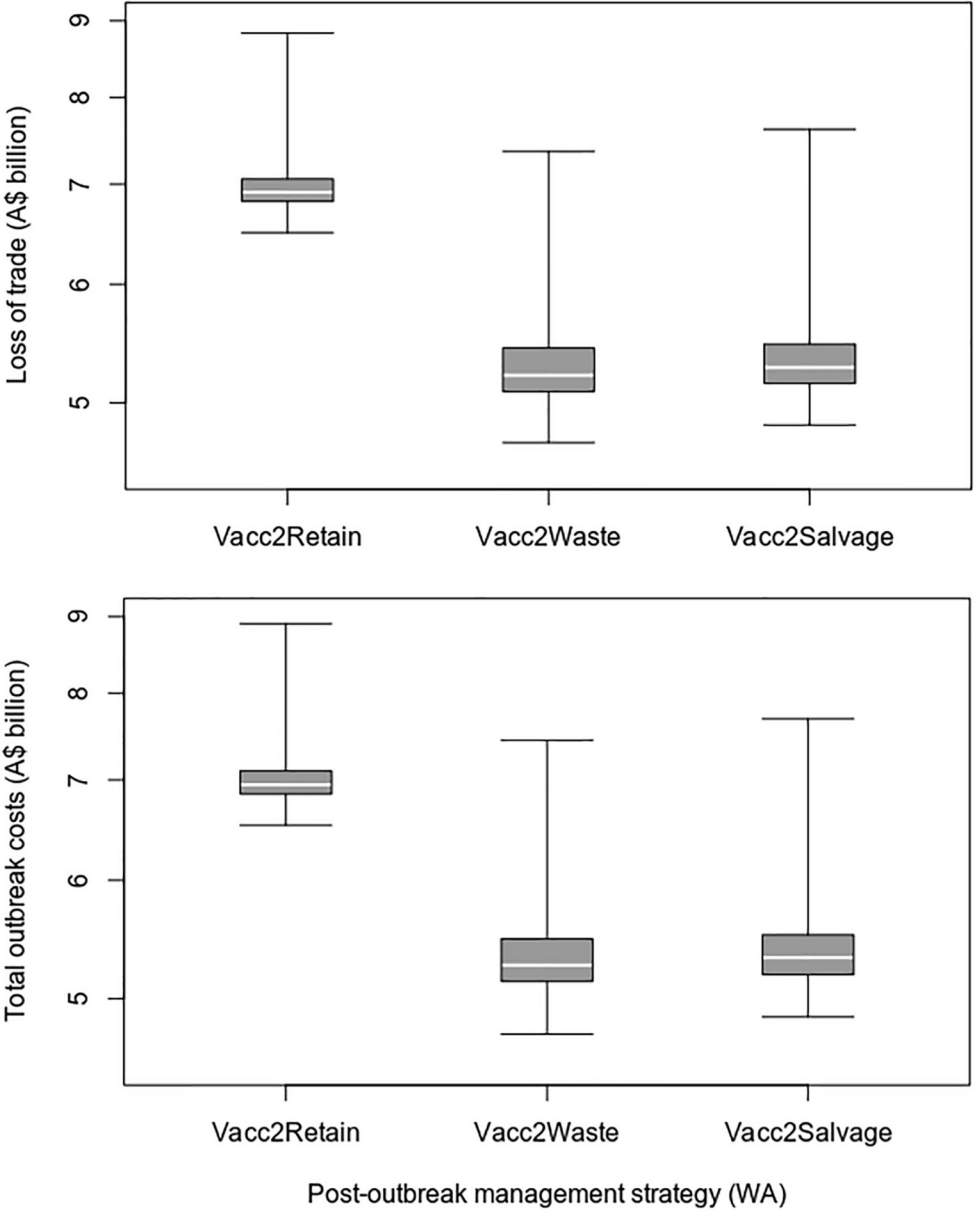
Loss of trade and total outbreak costs for different post-outbreak management strategies in the WA FMD outbreak scenario.

Under the assumptions used for this case study, the removal of vaccinated animals resulted in higher post-outbreak management costs and lower loss of trade costs, when compared to a vaccinate-and-retain strategy (Figs 7 and 8 and Table 11). On average, the vaccinate-and-remove-to-waste strategy resulted in 52 fewer days out of market and overall savings of A$1.66 billion (23.8%) compared to the vaccinate-and-retain strategy. There was no overall cost benefit from salvaging the removed vaccinated animals.

**Table 11:** Statistical summary of different post-outbreak management strategies for the WA FMD outbreak scenario.

Model outcome variable	Vaccinate-and-retain mean (95% CI)	Vaccinate-and-remove-to-waste mean (95% CI)	Vaccinate-and-remove-for-salvage mean (95% CI)
Number of IPs	43.8 (43.3, 44.4)^a^^†^	43.5 (43.0, 44.0)^a^	43.7 (43.2, 44.2)^a^
Number of VPs	49.1 (48.3, 49.8)^a^	48.4 (47.7, 49.2)^a^	49.0 (48.2, 49.8)^a^
Control program duration (days)	68.0 (67.6, 68.5)^a^	68.3 (67.8, 68.8)^a^	68.3 (67.8, 68.8)^a^
Surveillance costs (A$ million)	0.60 (0.59, 0.60)^a^	0.60 (0.59, 0.60)^a^	0.60 (0.59, 0.61)^a^
Culling, disposal and decontamination costs (A$ million)	2.40 (2.37, 2.43)^a^	2.39 (2.36, 2.42)^a^	2.40 (2.37, 2.43)^a^
Compensation costs (A$ million)	8.67 (8.56, 8.78)^a^	8.65 (8.54, 8.76)^a^	8.69 (8.58, 8.81)^a^
Vaccination costs (A$ million)	0.05 (0.05, 0.05)^a^	0.05 (0.05, 0.05)^a^	0.05 (0.05, 0.05)^a^
Control centre costs (A$ million)	23.03 (22.85, 23.23)^a^	22.86 (22.68, 23.04)^a^	22.91 (22.73, 23.09)^a^
**Total cost of control (A$ million)**	**34.85 (34.55, 35.16)**^**a**^	**34.66 (34.36, 34.95)**^**a**^	**34.76 (34.46, 35.06)**^**a**^
Post outbreak surveillance duration (days)	30.3 (30.0, 30.6)^a^	29.4 (29.1, 29.7)^b^	29.7 (29.4, 30.1)^b^
Post outbreak surveillance costs (A$ million)	1.70 (1.69, 1.72)^a^	1.69 (1.68, 1.71)^a^	1.71 (1.69, 1.73)^a^
Post outbreak laboratory costs (A$ million)	0.82 (0.81, 0.82)^a^	0.73 (0.73, 0.74)^b^	0.74 (0.73, 0.75)^b^
Post outbreak culling, disposal and decontamination costs (A$ million)	0.00 (0.00, 0.00)^a^	0.45 (0.44, 0.46)^b^	0.00 (0.00, 0.00)^a^
Post outbreak compensation costs (A$ million)	0.00 (0.00, 0.00)^a^	4.72 (4.62, 4.82)^b^	2.40 (2.35, 2.45)^c^
**Total post outbreak management costs (A$ million)**	**2.52 (2.49, 2.55)**^**a**^	**7.65 (7.53, 7.78)**^**b**^	**4.90 (4.83, 4.97)**^**c**^
Days out of market	217.5 (217.0, 218.0)^a^	165.4 (164.8, 166.0)^b^	167.1 (166.5, 167.7)^c^
Loss of trade (A$ million)	6960.66 (6943.98, 6977.38)^a^	5292.28 (5274.29, 5310.34)^b^	5348.31 (5329.26, 5367.42)^c^
**Total outbreak cost (A$ million)**	**6998.32 (6981.40, 7015.28)**^**a**^	**5335.01 (5316.71, 5353.38)**^**b**^	**5388.25 (5368.91, 5407.66)**^**c**^

^†^ Within rows, figures with differing superscripts are significantly different (p < 0.05)

## Discussion

The spread and control of FMD is a complex system with multiple virus serotypes, spread pathways and host species, compounded by heterogeneity in livestock production systems, marketing systems, geography and climate. Decision support tools such as the AADIS model can help unpack this complexity and allow policy makers to evaluate different control strategies in terms of effectiveness of disease control, and cost-effectiveness. Early detection of an incursion, effective control of an outbreak, and rapid return to trade are essential to minimise the economic impact of an outbreak in an FMD-free country. This is particularly important for a country such as Australia with large export-focused livestock industries. Australia’s policy for an FMD response is to contain, control and eradicate the disease in order to re-establish FMD-free status as quickly as possible, while minimising social and financial disruption. It is important for disease managers to also have clear guidelines on the post-outbreak management required to regain FMD-free status and facilitate the earliest possible return to trade. Policy should be based on sound epidemiological principles and take into account the nature of the outbreak, type of control program (particularly whether vaccination has been used or not), and economic implications of different management options.

There is increasing interest in the use of emergency vaccination in the control of FMD to avoid the need for large scale culling of animals [6]. However, under current international guidelines there is a significant economic disincentive to a vaccinate-and-retain policy because of the longer mandatory waiting period before FMD-free status can be regained, compared to removal of vaccinated animals from the population [4]. The disposal of healthy vaccinated animals just for the purpose of regaining markets has understandably been identified as an area of concern [17]. There is interest in the possibility of reducing the waiting period associated with a vaccinate-and-retain policy so as to align with vaccinate-and-remove policies [17].

The case studies presented in this paper demonstrate how the AADIS model can be used to quantify the effectiveness of vaccination as a control strategy, and the economic implications of how the vaccinated population is managed at the end of an outbreak. Augmenting stamping out with vaccination was highly effective in reducing the size and duration of the outbreak in the VIC case study, and provided a small but statistically significant improvement in the WA case study. The performance of control strategies in terms of containing and eradicating FMD depend on modelling assumptions such as the level of compliance with movement restrictions and the effectiveness of tracing, as well as the availability of resources needed to implement the measures. Vaccination is likely to offer advantages in terms of disease control over a stamping out only strategy when resources are inadequate to maintain effective surveillance and infected premises operations [7–9].

In both the VIC and WA case studies, the vaccinate-and-remove strategies were associated with higher post-outbreak management costs and lower loss of trade costs. In terms of overall outbreak cost, the vaccinate-and-remove-to-waste strategy provided average savings of around 8.7% (A$664 million) and 23.8% (A$1.66 billion) respectively in VIC and WA, compared to the vaccinate-and-retain policy. The greater benefits of a removal strategy in the WA scenario over the VIC scenario can perhaps be explained by the smaller outbreak sizes and fewer vaccinated premises. Assuming the same removal rates, the vaccinated population was removed to waste on average 31 days earlier in the WA scenario than the VIC scenario, leading to shorter periods until FMD-free status was regained. From a cost point of view there was no advantage with salvaging vaccinated animals as the potential salvage revenue was offset by the increased time out of market (stemming from the study assumption that it is quicker to remove animals to waste than salvage per Table 5).

The relative performance of vaccinate-and-remove compared to vaccinate-and-retain policies is influenced by the rate at which vaccinated animals can be removed. This is because the three-month waiting period before FMD-free status can be regained starts from when the last vaccinated animal is removed [4]. In the case of vaccinate-and-retain, the six-month delay until FMD-free status can be regained starts from when the last animal is culled or the last vaccination, whichever comes first [4]. For this study, we have conservatively estimated that 1000 cattle, 5000 sheep or 2000 pigs can be removed through abattoirs per day (Table 5). In a large outbreak, it could take many weeks or months to remove all the vaccinated animals. A simple sensitivity analysis of the removal rate was carried out to illustrate how the relative performance of a vaccinate-and-retain policy compared to vaccinate-and-remove policy is influenced by removal capabilities. For the Victorian case study, when the rates of removal were doubled, the average total outbreak costs under a vaccinate-and-remove-to-waste strategy fell 10% to A$6.28 billion, a further saving of A$1.36 billion compared to the vaccinate-and-retain approach. This suggests that if a vaccinate-and-remove policy is applied it will be important to remove vaccinated animals as quickly as possible. There will be no commercial reason for abattoirs to purchase and slaughter vaccinated animals so incentives/subsidies may need to be considered.

The calculation of trade losses in this case study was based on the minimum expected time to regain FMD free status under the OIE guidelines [4]. This is a very simplistic approach as it does not take into account the additional time required to negotiate the re-opening of closed markets and the recovery of market share [2]. It has been estimated that it could take up to 10 years for Australia to recover market share after an FMD outbreak [2]. The model user has the option of varying the compulsory waiting periods prior to the application for FMD-free status. Although the current modelling approach is adequate to explore relative differences between policies, more work is required to realistically estimate the actual time out of markets, taking into account different countries’ attitudes and approaches to FMD risk.

The OIE FMD code also now provides for establishment of a containment zone within an FMD free country [4]. Under this provision the free status of areas outside the containment zone are suspended while the containment zone is being established, but once the containment zone has been approved by the OIE, the free status of the non-infected areas may be reinstated. Although there is limited experience of this having been applied in previously FMD-free countries and accepted by trading partners, it offers significant potential for exporting countries like Australia to reduce the economic impact of an outbreak. Containment zones have been identified as an area for further research and is being addressed in another study.

The primary outcome of this study was a new modelling capability to quantify and compare the performance and economic impact of different approaches to post-outbreak management of vaccinated animals. This capability will support the development and refinement of Australian policy on FMD post-outbreak management that facilitates the earliest possible recovery of FMD-sensitive markets.

## Supporting information

S1 Table AADIS model outputs for the VIC and WA FMD outbreak scenario case studies. (ZIP)
